# Oral Mucositis: Melatonin Gel an Effective New Treatment

**DOI:** 10.3390/ijms18051003

**Published:** 2017-05-07

**Authors:** Ahmed Esmat Abdel Moneim, Ana Guerra-Librero, Javier Florido, Ying-Qiang Shen, Beatriz Fernández-Gil, Darío Acuña-Castroviejo, Germaine Escames

**Affiliations:** 1 Department of Zoology and Entomology, Faculty of Science, Helwan University, 11795 Cairo, Egypt; aest1977@hotmail.com; 2 Centro de Investigación Biomédica, Universidad de Granada, 18016 Granada, Spain; ana_guerra-librero@hotmail.com (A.G.-L.); jflorido@correo.ugr.es (J.F.); shenyingqiang@outlook.com (Y.-Q.S.); beatrizirenefg@correo.ugr.es (B.F.-G.); dacuna@ugr.es (D.A.-C.); 3 CIBERfes, Ibs.Granada, and UGC de Laboratorios Clínicos, Complejo Hospitalario de Granada, 18014 Granada, Spain

**Keywords:** mucositis, radiotherapy, chemotherapy, pathophysiology, management, melatonin

## Abstract

The current treatment for cervico-facial cancer involves radio and/or chemotherapy. Unfortunately, cancer therapies can lead to local and systemic complications such as mucositis, which is the most common dose-dependent complication in the oral cavity and gastrointestinal tract. Mucositis can cause a considerably reduced quality of life in cancer patients already suffering from physical and psychological exhaustion. However, the role of melatonin in the treatment of mucositis has recently been investigated, and offers an effective alternative therapy in the prevention and/or management of radio and/or chemotherapy-induced mucositis. This review focuses on the pathobiology and management of mucositis in order to improve the quality of cancer patients’ lives.

## 1. Overview of Mucositis Pathobiology

Mucositis, one of the most severe toxic side effects of cancer therapy, can affect the entire gastrointestinal tract, with the oral cavity being the most common affected site. It presents in virtually all head and neck cancer patients receiving chemo and/or radiotherapy, in 60–85% of those receiving myeloablative therapy for stem-cell transplantation, and in 20–40% of patients receiving conventional chemotherapy [1,2,3]. The use of concomitant chemotherapy and/or targeted agents increases the risk of mucositis.

Oral lesions lead to considerably decreased quality of life in these patients due to solid and liquid food dysphagia, dysarthria, and odynophagia, resulting in depression in some patients, who often require percutaneous endoscopic gastrostomy tube insertion [4]. In addition, mucositis lesions may represent a gateway for opportunistic infections, can complicate cancer treatment, and extend hospitalization [5,6]. On the other hand, given its dose-limiting toxicity for both chemo and radiotherapy, mucositis can have a direct impact on survival rates [7].

## 2. Pathophysiology of Mucositis

Recent developments in mucositis research have highlighted multiple factors which contribute to mucosal injury [8]. A five-phase chronological process has been proposed: initiation, primary damage response (upregulation and message generation), signal amplification, ulceration, and the healing phase [9]. Mucositis commences when gastrointestinal (GI) mucosa are exposed to cytotoxic agents, resulting in cellular DNA damage and cell death, mainly through the generation of oxidative stress and reactive oxygen species (ROS) formation. ROS directly induce tissue injury and trigger a cascade of inflammatory pathways [10]. Ortiz et al. have also observed a mitochondrial oxidation response to radiation with subsequent mitochondrial dysfunction [11].

The progression of mucositis is characterized by significant inflammatory mediator up-regulation due to the activation of the NF-κB pathway (upregulation and message generation phase). This is followed by the signaling and amplification phase, during which, once activated by chemotherapy and ROS, NF-κB promotes the expression of multiple pro-inflammatory molecules, including inducible nitric oxide synthase (iNOS), cyclooxygenase-2 (COX-2), TNF-α, and pro-IL-1β, and ensures feedback amplification of the NF-κB-dependent signaling pathway [10]. Furthermore, mitochondrial ROS production promotes inflammation by activating a multi-protein cytoplasmic complex, such as the NLRP3 (NACHT, LRR, and PYD domains-containing protein 3) inflammasome. NLRP3 inflammasome assembly leads to caspase-1 activation and subsequent cleavage of pro-cytokines such as pro-IL-1β, pro-IL-18, and pro-IL-33 into their mature form [11,12] resulting in ulceration (ulceration phase). Therefore, the NF-κB pathway, mitochondrial dysfunction, and subsequent NLRP3 inflammasome activation are the three main players involved in the development of oral mucositis, which amplify the whole inflammatory process via positive feedback loops, thus prolonging tissue injury and ending in the healing phase.

During the initiation phase of mucositis, patients begin to develop erythema followed by focal areas of oral mucosal desquamation [13], which mainly occur at the submucosa and basal epithelium level. Therefore, although no evident damage to mucosal integrity is observed clinically, the tissue biology is altered [10]. The progression of mucositis is then prolonged and severe, mucosal integrity is breached, ulceration begins to form and the patient starts to have a burning sensation. Atrophic changes occur in the GI mucosa, culminating in tissue injury and stem cell death. GI epithelial integrity and function are destroyed and impaired, respectively. A fibrinous exudate, or pseudomembrane, containing bacteria covers the ulcer. Bacterial colonization at the mucosa ulcers further induces inflammation by stimulating infiltration and activation of macrophages, which occurs approximately two weeks after therapy [10]. Cell wall residues originating from colonizing bacteria penetrate the submucosa, where they activate macrophages in the infiltrate [14]. This can lead to prolonged hospitalization, the need for parenteral nutrition, severe pain, risk of infection and sepsis, and increased risk of morbidity and mortality.

The final stage of mucositis pathobiology is the healing process. Epithelial cells controlled by signals secreted by the extracellular matrix, which are then downregulated to avoid hyperplasia, migrate, grow, and differentiate to form a wound. With the healing process under way, symptoms begin to abate [9], and healing is completed within 4 weeks after the final dose of radiation. Unfortunately, even after full replenishment of the epithelium, the structure of the reconstituted submucosa differs from its pre-radiotherapy state [15].

Intestinal mucositis is also a common side effect of anticancer chemotherapy that involves the small intestines. In intestinal mucositis, histopathological changes are also associated with the production of ROS and the amplification of inflammatory signals induced by anticancer drugs or radiation. Keefe et al. [16] studied patients undergoing chemotherapy with sequential duodenal biopsies pre and post treatment. They found that an increase in apoptosis was the first histological effect to be noted, with a seven-fold increase in apoptosis in intestinal crypts at day one post treatment. Reduction of the intestinal villous area, crypt length, and crypt proliferation then followed and the maximal effect was observed 3 days post treatment. Research is ongoing to fully elucidate the mechanisms of intestinal mucositis. The loss of crypt stem cells remains an important event in intestinal injury following anticancer treatment. However, it is not the sole contributing factor leading to overt damage. The inflammatory cascade is being realized as an important pathway in the development of intestinal mucositis that can be pharmacologically manipulated [17].

On the other hand, radiation-induced damage to healthy intestine tissue is a common side-effect caused by out-of-field or scattered radiation [18]. We recently demonstrated that tongue irradiation also induces intestinal damage. Typical macroscopic traces of mucositis were also detected in the small intestines of irradiated animals, including a significant decrease in villus height and morphological alterations associated with substantial intestinal architecture changes [19]. Given the involvement of mitochondrial oxidative stress, bio-energetic impairment, and subsequent NLRP3 inflammasome activation in the development of radiotherapy-induced gut toxicity, the oral irradiation of rats also resulted in increased small intestinal damage.

## 3. Mucositis Management

**Figure 1:**
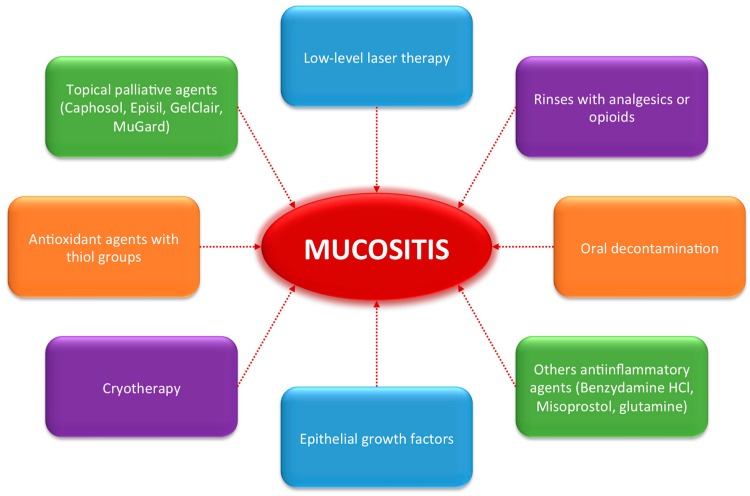
Actual strategies for managing oral mucositis.

As there is no effective therapy for mucositis or its associated pain, a large number of studies have been conducted in this field. Strategies for managing oral mucositis include preventative measures and therapeutic approaches (Figure 1) [13].

Current supportive measures to reduce the risk and severity of oral mucositis include improved oral hygiene, which eliminates the presence of any irritants to the oral mucosa [20]. Systemic analgesics are also recommended, as tissue injury activates nociceptive receptors which increase pain alongside the underlying tissue damage [7].

Several topical palliative agents, including Caphosol, Episil, GelClair, and MuGard, have been approved for the treatment of mucositis, are aimed at alleviating pain and improving the patient’s quality of life. However, data on the efficacy of these agents in controlling mucositis-related symptoms are scarce [13,21,22,23,24].

A combination of treatments, such as local rinses with a 2% viscous lidocaine solution, magic mouthwash preparations, a topical morphine solution, and other systemic analgesics are used to control pain [25]. Frequent rinsing with sodium chloride solution helps to keep the mucosa moist, reduces caking of secretions, and soothes inflamed/ulcerated mucosa. An oral rinse containing doxepin appears to be effective for easing acute oral mucositis pain caused by radiation therapy (with or without chemotherapy) [26]. However, there is no significant evidence to suggest that these mouthwashes are effective [27]. Another type of topical agent is the transdermal patch, composed of fentanyl, which is a potent, fast, and short acting synthetic opioid analgesic, although transdermal fentanyl causes mild dizziness, gastrointestinal reactions, and itching [28].

Oral decontamination, involving treatments such as Nystatin and chlorhexidine [29], may result in significant positive outcomes in the prevention of ulcerations linked to oral mucositis. Recently, Silva et al. [30] tested a soft pastille formulation consisting of 0.25% lidocaine and 78,000 IU Nystatin, which was found to facilitate accurate drug administration by physicians and to enable patients to control drug retention time in the mouth in order to manage the pain treatment process. On the other hand, chlorhexidine is an effective broad-spectrum antiplaque antiseptic agent [31]. However, these drugs are not very effective in reducing the severity or incidence of mucositis [32,33].

Cryotherapy, during which patients suck on ice chips for 30 min prior to and during chemotherapy infusion [34,35,36,37], has been shown to effectively attenuate the onset and severity of mucositis in patients undergoing chemotherapy with 5-fluorouracil and melphalan. However, it is only effective for short bolus chemotherapeutic infusions [38], may not be tolerated by some subjects, and thus cannot play a significant role in radiation-induced oral mucositis treatments [25].

Several antioxidant agents to prevent mucositis or to reduce its severity have been tested. One of the first drugs used to treat mucositis was amifostine [13], a thiol compound which is dephosphorylated to an active metabolite and acts as a potent ROS scavenger. However, given its limited and inconsistent results, amifostine is not recommended for the prevention of oral mucositis in patients receiving either chemotherapy or radiotherapy alone [39]. *N*-acetyl cysteine (NAC) is another antioxidant containing thiol groups, which stimulates glutathione synthesis and scavenges free radicals. In addition to its antioxidant properties, NAC prevents NF-κB activation which increases the inflammatory response. In a double-blind, randomized, placebo-controlled trial, NAC significantly reduced severe oral mucositis incidence [40].

Given that a reduction in the proliferative capacity of oral epithelial cells is thought to play a role in mucositis pathogenesis, various growth factors capable of increasing epithelial cell proliferation have been studied with regard to oral mucositis management. Although palifermin, an epithelial-specific growth factor, is the only agent approved for the prevention of oral mucositis in bone-marrow transplant patients [41], it is also associated with adverse side effects, requires intravenous administration, and is expensive [42]. Other growth factors, such as velafermin, filgrastim, and argramostim, have been analyzed for use in the treatment of mucositis [43]. Smad7, which has recently received considerable attention [44], was initially identified as a TGF-β superfamily signaling antagonist, which blocks TGF-β-induced growth inhibition and apoptosis in keratinocytes [45] and reduces inflammation by antagonizing NF-κB activation. All of these characteristics may make Smad7 beneficial in the treatment of oral mucositis [44].

Several anti-inflammatory agents have produced good results in studies of oral mucositis in animals; there is still conflicting evidence, however, on the efficacy of these agents in reducing the severity of mucositis in humans [13]. Benzydamine HCl is a non-steroidal anti-inflammatory drug that inhibits pro-inflammatory cytokines including TNF-α, and IL-1β. It has been administered in an intravenous formulation recommended for the prevention of oral mucositis in patients with head and neck cancer receiving moderate-dose radiation therapy without concomitant chemotherapy [46]. A long list of anti-inflammatory drugs has produced inconsistent results with regard to the prevention of chemotherapy-induced oral mucositis. This is the case for Misoprostol, a synthetic prostaglandin E1 analog, which has anti-inflammatory mucosa protection properties. However, the overall results of using misoprostol mouthwash in the prevention of radiation-induced oral mucositis in head and neck cancer patients were negative [47,48]. While glutamine, a nonessential amino acid, may mitigate mucosal injury by reducing pro-inflammatory cytokine production and cytokine-related apoptosis [49,50], it produced inconsistent results in the prevention of chemotherapy-induced oral mucositis [51,52,53].

Multiple studies have indicated that the application of low-level laser therapy (LLLT) reduces the incidence and, by hastening oral re-epithelialization, favorably influences oral mucositis outcomes in patients undergoing standard, hematopoietic stem cell transplantation and myeloablative chemotherapy [54,55,56,57,58,59]. Although the mechanism involved in these benefits is not understood, it has been suggested that LLLT may reduce ROS and/or pro-inflammatory cytokine levels which contribute to mucositis pathogenesis [25]. However, many of the pathways stimulated by LLLT are associated with undesirable tumor behaviors and/or treatment responses [60].

Moreover, there are some exciting approaches to the treatment and prevention of intestinal mucositis that could potentially be extended to the oral mucosa. These include probiotics, probiotic-derived factors, and plant extracts and preparations. A research study conducted by Yeung et al. [61] found that the daily oral administration of a probiotic suspension of Lactobacillus casei variety rhamnosus (Lcr35) or Lactobacillus acidophilus and Bifidobacterium bifidum (LaBi) resulted in the attenuation of diarrhea, proinflammatory cytokines, and jejunal villi damages in 5-FU(fluorouracil) -induced intestinal mucositis. Furthermore, a prospective open-labeled randomized trial in Sweden documented that *Lactobacillus rhamnosus* administration twice daily for 24 weeks reduced grade 3 or 4 diarrhea in patients and minimized the hospitalization due to bowel toxicity [62]. However, a major limitation of the Osterlund et al. [62] study was the lack of blinding and placebo control. A large prospective, double-blinded, placebo-controlled randomized trial at a single institution in Italy investigating the role of probiotics in the prevention of RT-induced acute diarrhea was conducted by Delia et al. [63]. The patients received a VSL#3^®^ sachet contained 4.5 × 10^11^/gram of viable lyophilized bacteria including four strains of *Lactobacilli*, three strains of *Bifidobacteria*, and one strain of *Streptococcus*. The authors found that significant improvements were noted in diarrhea. However, the overall methodology and results are very sparse in detail, limiting the reader’s ability to follow the trial.

Finally, traditional Chinese Medicine (TCM) offers empirical herbal formulas for treating mouth ulcers and stomatitis which has frequently been used in the complementary treatment of oral mucositis, but the evidence for these therapies is unclear and in some cases they have not been effective [64]. Also, the standard concepts for this kind of treatment do not exist and the acceptance by conventional oncologists is still low. In western complementary medicine, several herbal treatment approaches exist including *Salvia officinalis*, *Matriciana camomilla*, *Calendula officinalis*, *Hamamelis virginiana*, *Potentilla erecta*, *Commiphora molmol*, *Myrtilli fructus*, *Althaea spps*, *Malva spps*, *Cetraria islandica*, *Linum usitatissimum*, *Flos caryophylli*, *Hippophae rhamnoides*, *Aloe vera*, *Carica papaya*, *Centaurii herba*, *Gentianae radix*, *Menyanthes folium*, *Eriodictyon crassifolium*, *Oleum olivae* and *Citrus limon*. They are applied as single infusions for gargling or topical application [65]. Of these, *Matriciana camomilla*, *Salvia officinalis*, *Chimonanthus salicifolius*, *Aloe vera*, *Gentianae radix* and date palm pollen have been used [65,66,67,68,69]. The anti-mucositis activity of different herbs might be due to inhibiting apoptosis and inflammation in GIT (gastrointestinal tract). However, later phase III trials of *Matricaria chamomilla* have failed to conclude that the chamomile given in mouthwash formulations is effective in patients with chemotherapy-induced mucositis [70]. Table 1 summarizes the different agents used in mucositis management.

## 4. Melatonin: A New Treatment for Mucositis

Melatonin (*N*-acetyl-5-methoxytryptamine), a hormone synthesized from tryptophan, is produced by the pineal gland; it has been detected in multiple extrapineal organ tissues at much higher concentrations than in the pineal gland [78]. It is a potent free radical scavenger with anti-oxidant properties (Table 2), which increases the expression and activity of endogenous antioxidant enzymes such as superoxide dismutase (SOD), catalase, glutathione peroxidase (GPx), glutathione reductase (GRd), and γ-glutamyl-cystein synthase. This special class of antioxidant generates a series of metabolites that are also free radical scavengers when scavenging free radicals [79,80,81,82,83]. In other words, as compared to other antioxidants, melatonin is more effective in preventing damage caused by oxidative stress. Capable of crossing cell membranes and of easily reaching all cell compartments, it is taken up by mitochondria and can maintain mitochondrial homeostasis in different experimental models [78,84,85,86,87]. Melatonin increases membrane fluidity, electron transfer chain (ETC) complex activity, ATP production, and mitochondrial membrane potential, while reducing oxidative stress and closing mitochondrial permeability transition pores (MPTPs) [88]. Its important anti-inflammatory effects include expression inhibition of iNOS/i-mtNOS, COX-2, and pro-inflammatory cytokines such as IL-1β or TNF-α. Many of these properties are attributed to the inhibition of NF-κB-dependent innate immune pathway activation [89,90], and we recently showed that melatonin blunts NLRP3 inflammasome activation under different experimental conditions [11,91,92]. Table 2 summarizes the biological effects of melatonin.

### 4.1. Melatonin in the Oral Cavity

We recently demonstrated that melatonin is synthesized in oral mucosa, where it is involved in autocrine/paracrine signaling by binding to MT1 (melatonin receptor type 1A), MT2 (melatonin receptor 1B), and RORγ (RAR-related orphan receptor gamma) receptors, thus suggesting that it plays a role in normal oral mucosal physiology [11]. We found that aralkylamine *N*-acetyltransferase (AANAT) and acetylserotonin *O*-methyltransferase (ASMT), the two main enzymes in melatonin synthesis, are expressed in oral mucosa and also in salivary glands. However, melatonin, which is also found in saliva, is believed to passively enter the mucous through the circulatory system in salivary glands (parotid, submaxillary, and sublingual glands) [78,93]. Thus, as the quantity of melatonin entering the oral cavity is proportional to salivary flow, xerostomia, which actually aggravates periodontal status, could be associated with oral pathologies [94]. Together, these data, which are corroborated by the literature, demonstrate that melatonin has important implications for the prevention of oral cavity diseases by limiting tissue damage through free radicals and by stimulating immune responses. Almughrabi et al. demonstrated the relationship between low levels of melatonin in saliva and the increased severity of oral pathologies such as gingivitis, chronic periodontitis, and aggressive periodontitis [95]. The beneficial effects of topical melatonin applications in patients with periodontal diseases, as evidenced by improvements in the gingival index and pocket depth, have been established by Gomez-Moreno et al. [96]. Melatonin has also been proven to be an effective treatment for oral infections such as herpes [94]. Kara et al. showed that it decreases proinflammatory cytokines in gingivitis and periodontitis [97]. Cutando et al. [98] showed that melatonin treatment in patients with diabetes significantly reduced the gingival index, pocket depth, and RANKL (receptor activator of nuclear factor kappa-B ligand) levels, and also increased osteoprotegrin concentrations. Due to its anti-inflammatory and anti-oxidative effects, the severity of gingival and periodontal inflammation was reduced. Other studies have demonstrated that melatonin could be a beneficial therapy after surgical procedures in the oral cavity by preventing inflammatory and infectious complications induced by oxidative stress [99]. Cutando et al. [99] showed that topically applied melatonin in the evacuated sockets following tooth removal reduced oxidative stress and inflammation and accelerated the healing process. Thus, by being directly deposited in the oral cavity, it has the capacity to treat oral disorders and pathologies by reducing inflammatory responses in the gingiva and periodontium [94].

### 4.2. Melatonin as a Radio-Protective Agent

For many years, the radio-protective effects of melatonin have been observed in different experimental models as well as organs and tissues. Tan et al. [113] were the first to report its ability to protect against electromagnetic radiation emitted by ultraviolet light, with subsequent studies reporting its effectiveness in protecting against ionizing radiation. Vijayalaxmi et al., who carried out a series of experiments to study the radio-protective effects of melatonin in vitro and in vivo, showed that melatonin guarded against γ radiation-induced cell damage in blood lymphocytes [114,115,116,117]. Other authors have reported that it prevents hemolysis in irradiated human red blood cells [118]. Pretreatment with melatonin guards erythrocytes, granulocytes, macrophages, megakaryotes, and T cells against radiation-induced cellular injury and also inhibits splenocyte apoptosis in whole-body irradiated mice [119]. It also prevents radiation-induced damage in retinal cells [120], thymocytes [121], and bone-marrow cells in mice [122]. Several in vivo studies reveal that it increases the survival rate of animals exposed to radiation. Pre-treatment with 250 mg/kg melatonin raised the survival rate of lethally whole-body irradiated (9.5 Gy) mice [123] to approximately 43% and to 85% at a dose of 8.15 Gy [124]. Iwata et al. demonstrated that pre-treatment with intraperitoneally administered melatonin at a dosage of 150 mg/kg showed a survival rate of around 100% for mice following a radiation dose of 7.5 Gy [125], with an amelioration in radiation-induced injury in radio-sensitive organs such as bone marrow, spleen, and gastrointestine [126]. In all these experiments, melatonin was observed to protect against radiation-induced genotoxicity in both the somatic and germ cells of mice. Its radio-protective impact was also demonstrated in the testis and ovary of irradiated rodents [127,128,129,130]. Melatonin also plays an important protective role against radiation-induced damage to the thyroid gland [131] and the small intestine [132], which is one of the most radio-sensitive organs, resulting in inflammation-induced radiation enteritis. Its administration prior to irradiation guards against intestinal damage caused by X-rays [133]. It prevents mucosal intestinal damage caused by radiotherapy by countering structural changes in the small intestine (as evidenced by the villous pattern of the intestinal mucosa) [134] and also inhibits gut bacterial translocation to the spleen, liver, and kidney [119].

The mechanisms involved in melatonin as a radioprotective agent have been attributed to its antioxidant properties, which reduce radiation-induced DNA damage and lipid peroxidation, and to its protection of the immune system by reducing apoptosis through the inhibition of p53 and Bax and by enhancing anti-apoptotic protein Bcl-2 [119,126,135]; it is also involved in repairing lesions in cellular DNA [136].

In patients, the administration of melatonin resulted in amelioration of hypotension, myelotoxicity, and lymphocytopenia associated with radiotherapy [137]. Therefore, its use as a prophylactic agent could reduce morbidity and limit radiation-induced injury in cancer patients under radiotherapy [119,135].

### 4.3. The Use of Melatonin in the Prevention of Radiation-Induced Mucositis

Together, given the data summarized in this review, highlighting its anti-inflammatory and antioxidant function in the oral cavity and its potential effectiveness in protecting against ionizing radiation, melatonin could, in our view, play a beneficial role in the prevention of mucositis. We demonstrated that the application of a melatonin gel to oral mucosa prevented oral mucositis in irradiated rats [11]. For these experiments, the tongue was irradiated under anesthesia (1 mL equithesin/kg body weight, IP) at 7.5 Gy/day for 5 consecutive days. Melatonin gel or vehicle was applied in the oral cavity 48 h before each irradiation dose and up to 14 days after the last irradiation exposure. Melatonin gel was applied three times a day in the intraoral regions using a plastic Pasteur pipette. The gel containing 3% melatonin was dissolved in 0.3% ethanol:Pluronic F-127. The gel was made in our laboratory and it is under patent. Previously, we performed a dose-response study and the maximal therapeutic effect was obtained with 3% melatonin gel. Therefore, in the group that received melatonin gel, the total melatonin dose was 45 mg/day for 21 days. The purpose of this study was to investigate the pathophysiology of oral mucositis and how melatonin can prevent its development. We observed that melatonin gel is capable of preventing mucosal disruption and the emergence of ulcers. Furthermore, it can prevent the loss of proliferative progenitor stem cells caused by radiation and enhance their capacity to repopulate the tissue. In addition, ionizing radiation exposure causes oxidative damage to DNA, which arrests the cell cycle, inhibits growth, and increases cell death [119]. Thus, the protection of cellular DNA is of utmost importance in reducing radiation-induced cellular perturbation and also in the proliferation/differentiation of normal cells. Melatonin treatment significantly reduces radiation-induced DNA degradation. Therefore, given the high dependence of both epidermis and mucosal epithelia on resident self-renewing stem cells, the therapeutic interventions using melatonin described above, which can reduce the deleterious effects of radiation on normal epithelial stem cells, could have a considerable impact on the quality of life of cancer patients.

**Figure 2:**
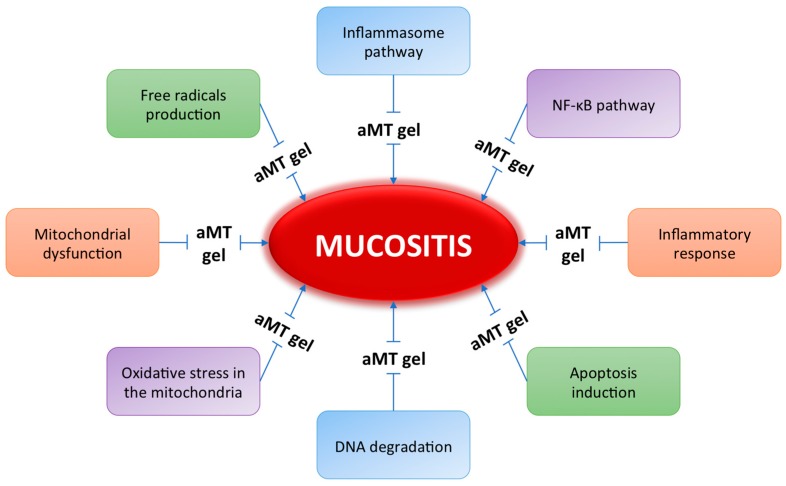
Molecular pathways of melatonin gel for preventing oral mucosa.

On the other hand, it is well known that irradiation inhibits the activity of mitochondrial ETC complexes which enhances electron leakage and subsequent superoxide anion (O_2_^• −^) generation, leading to persistent oxidative stress, responsible, at least partially, for radiation-induced cell death in normal human fibroblast cells [138]. Given the induction of mitochondrial dysfunction by ionizing radiation, we assessed whether mitochondrial damage is involved in radiation-induced mucositis. We also determined whether the known improvement by melatonin of mitochondrial function could protect oral mucosa against deleterious radiation effects. In our experiments, we found that the application of melatonin gel decreased the radiation-induced oxidation responses of mitochondria. We also observed that the protective effect of melatonin was mediated by the increased expression and activity of mitochondrial antioxidant enzymes such as GRd. This reduced the mitochondrial GSSG/GSH (oxidized glutathione/reduced glutathione) ratio and restored mitochondrial GSH homeostasis [84], thus enabling mitochondria to recover from post-radiation oxidative stress. This is in line with the finding that melatonin gel increases ETC protein expression and activity and also expands citrate synthase activity, reflecting the increase in mitochondrial mass, resulting in a full recovery of their bio-energetic capacity and the prevention of cell death. These findings suggest that the reduction in mitochondrial damage by melatonin by preserving mitochondrial structure and function may underlie its efficacy in preventing mucositis. The prevention of mitochondrial damage in irradiated oral mucosa may therefore constitute one of the mechanisms by which melatonin protects against cell death. Interestingly, we found that it inhibits mitochondrial-dependent apoptosis by decreasing the Bax/Bcl-2 ratio. It also significantly inhibits p53 and Bax protein expression, while anti-apoptotic Bcl-2 protein expression increases considerably. Consequently, at least part of its radio-protective capacity could depend on the inhibitory action of p53-related signaling proteins, which prevents the opening of MPTPs and thus blocks the release of cytochrome c to the cytosol. In this regard, radiation induces MPTPs to open up and mitochondrial DNA (mtDNA) to translocate to the cytosol. In addition to ROS released from damaged mitochondria to the cytosol, mtDNA promotes the assembly of NLRP3 inflammasome, a multiprotein complex which activates caspase-1 [139]. Once activated, caspase-1 converts NF-κB-dependent pro-inflammatory cytokines, including pro-IL-1β, into a mature form. Thus, the activation of both NLRP3 inflammasome and NF-κB innate immunity pathways leads to the overproduction of IL-1β, TNF-α, and other pro-inflammatory mediators [139]. This double-stranded, innate immune response by the NF-κB and NLRP3 inflammasome pathways could explain the difficulty of finding an effective anti-mucositis therapy. Furthermore, the presence of impaired mitochondria underlying these inflammatory responses makes the search for a specific treatment more difficult. Our results highlight the importance of scavenging ROS and the protection of mitochondria from irradiation in order to suppress inflammasome activation and consequently the development of oral mucositis. This would, in turn, help to explain the therapeutic benefits of melatonin gel in combating oral mucositis (Figure 2).

On the other hand, as discussed above, epithelial crypts in the intestinal region contain the highest proliferating cells [140], which are extremely sensitive to ionizing radiation [141]. Recent studies have reported that, even when the intestine is outside the irradiation field, radiation-induced damage to healthy intestine tissue is a common side-effect of out-of-field or scattered radiation. This could explain why typical signs of mucositis were detected in the small intestine following tongue irradiation. Histological analysis revealed crypt loss, lower villi numbers, and shorter lengths in the gastrointestinal region, leading to the conclusion that radiation enteropathy prevention requires protection of the small intestine. Melatonin gel treatment in the mouth, which was observed to reduce intestinal morpho-pathological changes [19], is therefore also associated with improved preservation of both small intestinal and oral mucosa.

In addition, a number of studies have shown that melatonin contains remarkable oncostatic properties. Its antiproliferative properties have been demonstrated in an extensive variety of tumors, including breast, endometrial, prostate, colon, and ovarian cancers as well as choriocarcinoma, melanoma, neuroblastoma, and osteosarcoma among others [142,143]. Its oncostatic mechanisms are associated with several hallmarks of cancer. Its anticancer action directly inhibits the proliferation and growth of tumor cells [144]. Melatonin protects normal cells from apoptosis, while, at the same time, promoting apoptotic cell death in several types of cancer cells [145]. Its immune-modulatory anticancer action also augments antitumor immune response [146]. Additionally, melatonin has an important metabolic effect, which decreases glucose uptake by cancer cells [147] and inhibits tumor growth through the suppression of the uptake of linoleic acid and its metabolism by the tumor to the mitogenic molecule 13-HODE [148]. Its anti-angiogenic and anti-metastatic properties have also been reported in numerous studies [149,150,151]. Given its capacity to increase the efficacy of anticancer drugs, these data show that melatonin can be used not only to treat mucositis due to the absence of adverse side effects, but also can be used in cancer co-treatment programs.

## 5. Conclusions

In summary, oral mucositis is a clinically important, deleterious consequence of chemo and radiotherapy, for which no effective treatment has been found to date. Mucositis lesions can be painful, affect nutrition and quality of life, and have a significant economic impact. The pathogenesis of oral mucositis is multifactorial and complex, and not all mucositis can be prevented. Once mucositis has developed, therapy should focus on supportive care, which aims to maintain hydration, provide appropriate caloric intake through enteral or parenteral nutritional support, relieve pain, and to prevent infection. This review discusses current clinical practices in the management of oral mucositis and emphasizes that no standard therapeutic approach has been developed for patients suffering from oral mucositis. Thus, basic, translational, and clinical research into how to prevent and treat oral and gastrointestinal mucositis continues. Our findings, showing that melatonin reduces irradiation toxicity and prevents treatment-induced mucositis, indicate that, with its oncostatic and cytoprotective properties, it constitutes an innovative, adjuvant strategy in the treatment of cancer. We demonstrated that treatment with melatonin gel protects rats from post-radiation oral mucositis, prevents duodenal inflammation and necrosis, and restores mucosal endogenous melatonin levels in irradiated animals. A clinical trial of this gel, which is under patent, is currently underway to test for the prevention of oral mucositis in head and neck cancer patients.

## Figures and Tables

**Table 1:** Studies which evaluated the effect of different agents on the management of mucositis.

Agent	Experiment	Subject	Outcomes	Reference
Mouth rinse				
Traumeel S	A randomized, controlled clinical trial of the homeopathic medication Traumeel S in the treatment of chemotherapy-induced stomatitis in children undergoing stem cell transplantation	Human	The severity and duration of chemotherapy-induced stomatitis were reduced	[71]
Topical agents				
MuGard	Multi-institutional, randomized, double-blind, placebo-controlled trial to assess the efficacy of a mucoadhesive hydrogel (MuGard) in mitigating oral mucositis symptoms in patients being treated with chemoradiation therapy for cancers of the head and neck	Human	MuGard lessened the severity of the developing mucositis and pain	[21]
Fentanyl	Efficacy and safety of transdermal fentanyl for the treatment of oral mucositis pain caused by chemoradiotherapy in patients with esophageal squamous cell carcinoma	Human	Fentanyl was effective in treating pain from oral mucositis caused by chemoradiotherapy	[28]
Prophylaxis and decontamination				
Nystatin	Efficacy of chlorhexidine and nystatin rinses in the prevention of oral complications in leukemia and bone marrow transplantation	Human	Nystatin rinse has not been found to be effective in reducing the severity of chemotherapy-induced mucositis	[32]
Antioxidants				
Amifostine	Chemotherapy- and radiotherapy-induced oral mucositis: Review of preventive strategies and treatment	Human	Amifostine may reduce the frequency of severe esophagitis in patients undergoing concomitant chemotherapy and radiotherapy for non–small cell lung cancer	[72]
*N*-acetyl cysteine (NAC)	*N*-acetyl cysteine for the prevention of oral mucositis in hematopoietic sct: A double-blind, randomized, placebo-controlled trial	Human	NAC significantly reduced the incidence of severe oral mucositis (grades 3–4) after high-dose chemotherapy and no patient in the NAC group developed grade 4 mucositis	[40]
*Matricaria chamomilla*	Prospective evaluation of a chamomile mouthwash for the prevention of 5-FU-induced oral mucositis	Human	Later phase III trials of *Matricaria chamomilla* have failed to conclude that the chamomile given in mouthwash formulations is effective in patients with chemotherapy-induced mucositis	[70]
Growth factors				
Keratinocyte growth factor (KGF)	Palifermin for oral mucositis after intensive therapy for hematologic cancers	Human	KGF significantly reduced the incidence of grade 3 and 4 oral mucositis in patients with hematologic malignancies	[41]
Recombinant human epidermal growth factor (rhEGF)	Recombinant human epidermal growth factor treatment of radiation-induced severe oral mucositis in patients with head and neck malignancies	Human	rhEGF has been shown to enhance the mucosal wound healing process and has a therapeutic effect on radiation-induced oral mucositis	[73]
Anti-inflammatory agents				
Glutamine	Oral glutamine reduces the duration and severity of stomatitis after cytotoxic cancer chemotherapy. Oral glutamine to alleviate radiation-induced oral mucositis: A pilot randomized trial	Human	In two small, randomized studies prophylactic glutamine mouthwashes significantly reduced the incidence, severity, and duration of oral mucositis in patients undergoing radiotherapy or chemotherapy, respectively	[74,75]
Locally applied nonpharmacological agents				
Low level laser therapy (LLLT)	Cyclooxygenase-2 and vascular endothelial growth factor expression in 5-fluorouracil-induced oral mucositis in hamsters: evaluation of two low-intensity laser protocols	Hamster	LLLT promotes wound healing and appears to have an anti-inflammatory effect, as evidenced by the reduction in neutrophil infiltrate	[76]
	Phase III trial of low-level laser therapy to prevent oral mucositis in head and neck cancer patients treated with concurrent chemoradiation	Human	Preventive LLLT in HNSCC (head and neck squamous cell carcinoma) patients receiving chemoradiotherapy is an effective tool for reducing the incidence of grade 3-4 oral mucositis	[77]

**Table 2:** Melatonin biological effects. Up arrows (increase) whereas, down arrow (decrease).

Molecule, Activity, or Process	Biological Effect of Melatonin	References
Reactive oxygen species		
OH^• −^ (hydroxyl radical)	↓	[100]
O_2_^• −^ (oxygen free radical)	↓	[100]
H_2_O_2_ (hydrogen peroxide)	↓	[83]
LO^• −^, LOO^• −^ (alkoxyl, peroxyl radicals)	↓	[101]
NO (nitric oxide)	↓	[83]
ONOO^• −^ (peroxynitrite)	↓	[102]
DNA lesions		
8-hydroxyguanine	↓	[103,104]
8-oxo-2′-deoxyguanosine	↓	[103,104]
Inflammation		
NF-κB (nuclear factor-κB)	↓	[11,91,92]
COX-2 (cyclooxygenase-2)	↓	[105]
Interleukins	↓	[106,107]
NLRP3	↓	[11,91,92]
TNF- α (tumor necrosis factor-α)	↓	[106,107]
iNOS (inducible nitric oxide synthase)	↓	[106,107]
MPO (myeloperoxidase)	↓	[108]
Cell death		
p53	↓	[109]
Caspases (Cas-3, 8, 9, …)	↓	[11,83]
cytochrome *c* (in cytosol)	↓	[109]
Bcl-2, Bcl-xL (anti-apoptosis)	↓	[11,83]
Bax, Bak (pro-apoptosis)	↓	[11,83]
Autophagy		
Beclin-1, Atg3, Atg12, …. (pro-autophagy)	↓	[110,111]
mTOR (pro-autophagy)	↓	[110,111]
PI3K/Akt (anti-autophagy)	↑	[110,111]
Antioxidative defense system		
GSH (glutathione)	↑	[11,83]
SOD (superoxide dismutase)	↑	[11,83]
CAT (catalase)	↑	[11,83]
GPx (glutathione peroxidase)	↑	[11,83]
GRd (glutathione reducatse)	↑	[11,83]
glutathione synthetase	↑	[112]
γ-glutamyl-cysteinyl synthetase	↑	[112]

## Abbreviations

ApoE	Apolipoprotein E
COX-2	Cyclooxygenase-2
CTCAE	Common terminology criteria for adverse events
EGF	Epidermal growth factor
G-CSF	Granulocyte colony-stimulating factor
GIT	Gastrointestinal tract
GM-CSF	Granulocyte-macrophage colony stimulating factor
IL-1	Interleukin-1
MAPK	Mitogen-activated protein kinase
MMP	Matrix metalloproteinase
mTOR	Mammalian target of rapamycin
NAC	*N*-acetyl cysteine
NCI	National Cancer Institute
NF-κB	Nuclear factor-κB
NLRP3	NACHT, LRR, and PYD domains-containing protein 3
NSAID	Non-steroidal anti-inflammatory drug
PARs	Protease-activated receptors
PGE	Prostaglandin E
PLAG	1-palmitoyl-2-linoleoyl-3-acetyl-rac-glycerol
rhEGF	Recombinant human EGF
ROS	Reactive oxygen species
SAP	Serum amyloid-P
Smad7	Mothers against decapentaplegic homolog 7
TCM	Traditional Chinese medicine
TGF-β	Transforming growth factor-β
TNF-α	Tumor necrosis factor-α
